# MQADet: a plug-and-play paradigm for enhancing open-vocabulary object detection via multimodal question answering

**DOI:** 10.1038/s41598-026-36936-x

**Published:** 2026-01-27

**Authors:** Caixiong Li, Xiongwei Zhao, Jinhang Zhang, Xing Zhang, Qihao Sun, Zhou Wu

**Affiliations:** 1 School of Computer and Information Science, Qinghai Institute of Technology, Xining, 810016 China; 2 Qinghai Provincial Key Laboratory of Big Data in Finance and Artificial Intelligence Application Technology, Xining, 810016 China; 3 School of Information Science and Technology, Harbin Institute of Technology (Shen Zhen), Shenzhen, 518055 China; 4 State Key Laboratory of Robotics and System, Harbin Institute of Technology, Harbin, 150000 China; 5 School of Computer Science and Technology, Qinghai University, Xining, 810016 China; 6 Eryuan Digital Technology Co., Ltd., Zhengzhou, 450000 China

**Keywords:** Open-vocabulary detection, Multimodal question answering, Multimodal large language models, Engineering, Mathematics and computing

## Abstract

Open-vocabulary detection (OVD) aims to detect and classify objects from an unrestricted set of categories, including those unseen during training. Existing open-vocabulary detectors often suffer from visual-textual misalignment and long-tailed category imbalance, leading to poor performance when handling objects described by complex, long-tailed textual queries. To overcome these challenges, we propose Multimodal Question Answering Detection (MQADet), a universal plug-and-play paradigm that enhances existing open-vocabulary detectors by leveraging the cross-modal reasoning capabilities of multimodal large language models (MLLMs). MQADet can be seamlessly integrated with pre-trained object detectors without requiring additional training or fine-tuning. Specifically, we design a novel three-stage Multimodal Question Answering (MQA) pipeline that guides MLLMs to accurately localize objects described by complex textual queries while refining the focus of existing detectors toward semantically relevant regions. To evaluate our approach, we construct a comprehensive benchmark across four challenging open-vocabulary datasets and integrate three state-of-the-art detectors as baselines. Extensive experiments demonstrate that MQADet consistently improves detection accuracy, particularly for unseen and linguistically complex categories, across diverse and challenging scenarios. To support further research, we will publicly release our code.

## Introduction

**Fig. 1:**
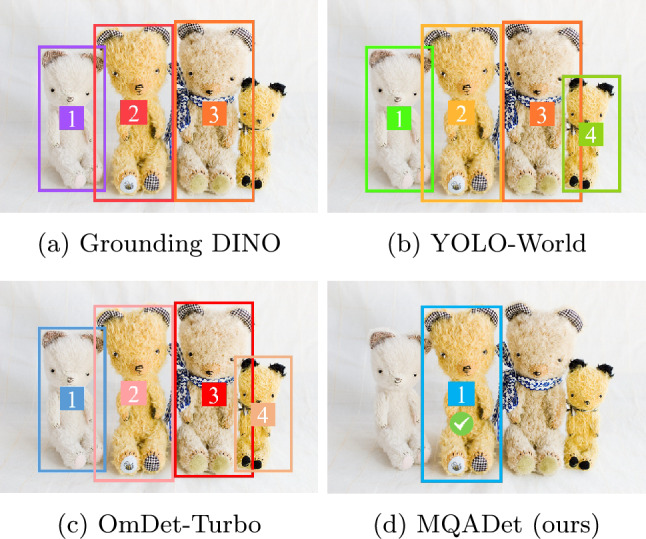
Comparison of existing open-vocabulary detectors and the proposed MQADet under a challenging textual query: *“a teddy bear with a checkered design on one foot and a bumble bee design on the other foot. the bear also has the checkered design over its ’ ears”* Grounding DINO, YOLO-World, and OmDet-Turbo produce multiple candidate boxes and do not identify the correct instance. MQADet localizes the intended object based on the provided description, indicating improved robustness for linguistically complex queries.

Object detection is a fundamental task in computer vision and serves as a cornerstone for numerous applications, including image analysis, robotics, and autonomous driving^1–3^. Recent advances in deep learning architectures have led to remarkable improvements in detection accuracy across various benchmarks^4–6^. However, most conventional detectors remain inherently limited by a fixed set of predefined categories, such as the 80 classes in the COCO dataset^7^. These models can only recognize the object categories they were explicitly trained on, and extending them to novel concepts typically requires large-scale human annotation and labor-intensive retraining procedures. With the proven reasoning capabilities of multimodal large language models (MLLMs) in visual-linguistic tasks^8,9^, recent research^10–12^ has sought to extend these capabilities to open-vocabulary (OV) detection. While such approaches have achieved notable progress, they still face two major challenges. First, their ability to align complex visual and textual information remains insufficient. As illustrated in Figure 1, when tasked with detecting objects in multi-instance scenes described by complex textual queries–such as “a teddy bear with a checkered design on one foot and a bumble bee design on the other foot. the bear also has the checkered design over its ’ ears”–state-of-the-art OVD methods, including Grounding DINO^10^, YOLO-World^11^, and OmDet-Turbo^12^, fail to accurately localize the queried teddy bear. This failure highlights the difficulty of current models in interpreting linguistically intricate descriptions and establishing effective visual-textual correspondence, which limits their reasoning ability across diverse attributes. Second, these methods demand substantial computational resources and retraining costs, limiting their scalability and real-world applicability.

To address these challenges, we propose Multimodal Question Answering Detection (MQADet), a plug-and-play paradigm designed to enhance the performance of existing open-vocabulary detectors in handling complex textual queries without requiring any additional training. MQADet adopts a three-stage Multimodal Question Answering (MQA) pipeline: (1) Text-Aware Subject Extraction (TASE) leverages the advanced linguistic understanding of MLLMs to extract key subjects and corresponding attributes from complex textual descriptions; (2) Text-Guided Multimodal Object Positioning (TMOP) employs these extracted cues to guide existing OV detectors toward precise object localization; and (3) MLLMs-Driven Optimal Object Selection (MOOS) performs fine-grained reasoning to ensure accurate visual-textual alignment and optimal object selection. This hierarchical design effectively mitigates the challenges of textual complexity and misalignment, substantially improving detection accuracy under open-vocabulary settings. In summary, the main contributions of this work are as follows:

- We propose MQADet, a plug-and-play paradigm that seamlessly integrates with pre-trained detectors without additional training overhead. It introduces a three-stage Multimodal Question Answering (MQA) pipeline that substantially enhances open-vocabulary detection performance, particularly when handling complex and descriptive textual queries.
- We establish a comprehensive benchmark to evaluate MQADet across four challenging open-vocabulary datasets, integrating three representative OVD baselines. A detailed analysis and comparison are further provided, yielding valuable insights into MQADet’s generalization ability across diverse query types and object categories.
- Extensive experiments demonstrate that MQADet consistently improves detection accuracy, achieving average gains of 13% on RefCOCO, 9% on RefCOCO+, 20% on RefCOCOg, and 27% on Ref-L4. These results confirm the robustness and effectiveness of MQADet in tackling complex textual reasoning and visual-textual alignment challenges.

## Related work

### Open-vocabulary detection

Open-vocabulary detection (OVD) seeks to generalize beyond a limited set of annotated base classes and detect arbitrary novel categories in the wild. CLIP^13^ employs cross-modal contrastive learning on large-scale image-text datasets to align image and text embeddings within a shared latent space, enabling effective zero-shot transfer to OVD tasks. ViLD^14^ utilizes visual-linguistic knowledge distillation to transfer the representation ability of CLIP into a two-stage detector, thereby improving zero-shot detection performance. Region-CLIP^14^ extends CLIP to learn region-level visual representations, thereby enhancing its ability to handle open-set detection tasks. Grounding DINO^10^ builds upon self-supervised learning principles and adopts a tightly coupled modality fusion design based on DINO^15^, achieving improved zero-shot generalization through large-scale grounded pre-training. YOLO-World^11^ introduces a Re-parameterizable Vision-Language Path Aggregation Network (RepVL-PAN) and a region-text contrastive loss to improve cross-modal interaction while maintaining high performance with reduced computational cost. These approaches typically employ single-stage fusion frameworks with lightweight architectures, aiming to achieve end-to-end vision-language understanding. However, they struggle to achieve fine-grained alignment between complex textual descriptions and visual representations, which hinders zero-shot transfer and weakens language generalization. Such models often underperform on unseen datasets, particularly when interpreting long and linguistically complex textual queries.

### Modality information fusion

Effective open-vocabulary detection fundamentally depends on robust multimodal information fusion and precise alignment between visual and linguistic modalities. CLIP^13^ aligns entire images with textual descriptions but lacks the capacity to capture fine-grained region-text correspondences. MEDet^16^ and VL-PLM^17^ achieve region-text alignment by introducing region proposal networks (RPNs) or class-agnostic proposal generators, typically using single-word category representations. However, these methods fail to capture the semantics of long and complex sentences, which remains a major obstacle to achieving nuanced vision-language alignment. CoOp^18^ observes that subtle variations in textual prompts can significantly influence the performance of vision-language pre-training models. It introduces a context-optimization mechanism for automatic prompt representation learning in pre-trained vision-language models. DetPro^19^ integrates CoOp into open-vocabulary detection, enabling prompt representation learning based on positive and negative proposal sampling. TaskCLIP^20^ employs a two-stage architecture combining general object detection with VLM-guided object selection. It further refines cross-modal alignment through a transformer-based aligner that recalibrates embeddings across visual and textual modalities. Despite their effectiveness, these methods demand complex training and high computational costs, which hinder scalability and real-world deployment.

Recent multimodal attribute recognition methods, such as CLEAR^21^ and C2T-Net^22^, improve vision-language alignment through transformer-based cross-modal fusion. However, these approaches rely on task-specific supervision and limited-domain data. In contrast, MQADet is a training-free, plug-and-play paradigm that leverages MLLMs to enhance open-vocabulary detectors and align complex textual queries with visual targets.

## Method

### Problem formulation

In this work, MQADet aims to identify the optimal target objects from complex textual queries by integrating multimodal large language models (MLLMs) with existing open-vocabulary (OV) detectors via a Multimodal Question Answering (MQA) framework. Given a user query text composed of *n* tokens, denoted as $T = \{w_1,..., w_n\}$, the query may contain object categories, noun phrases, or descriptive attributes. The MLLMs first extract the object subjects from the textual input, denoted as $\{OS_i\}_{i=1}^{M}$, where *M* is the number of subject entities identified in the query. The extracted subjects, together with the input image *I*, are fed into the open-vocabulary detectors *Dets* to generate candidate bounding boxes $\{Boxes_i\}$ and their corresponding object marks $\{Marks_i\}$, forming a marked image *MI*. Finally, MLLMs are employed to align the textual query *T* with the marked image *MI*, yielding the optimal detection results *OB*.

**Fig. 2:**
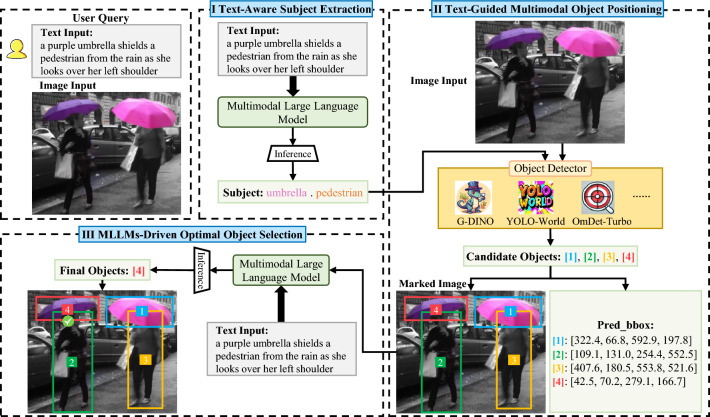
Overview of the proposed MQADet paradigm, comprising three Multimodal Question Answering (MQA) stages: (1) Text-Aware Subject Extraction (TASE), (2) Text-Guided Multimodal Object Positioning (TMOP), and (3) MLLMs-Driven Optimal Object Selection (MOOS). The numeric labels are rendered in white, 16-point font, centered inside the candidate bounding boxes to ensure accurate recognition by MLLMs. The final detected object in this example is the purple umbrella (object 4).

### Architecture of MQADet

MQADet is a plug-and-play paradigm designed to enhance open-vocabulary detection (OVD) without additional pre-training. It offers a new perspective for addressing complex visual-textual alignment challenges by harnessing the visual perception and cross-modal reasoning capabilities of multimodal large language models (MLLMs). The MQADet framework comprises three Multimodal Question Answering (MQA) stages: Text-Aware Subject Extraction (TASE) (Section 3.3), Text-Guided Multimodal Object Positioning (TMOP) (Section 3.4), and MLLMs-Driven Optimal Object Selection (MOOS) (Section 3.5), as shown in Figure 2.

**Fig. 3:**
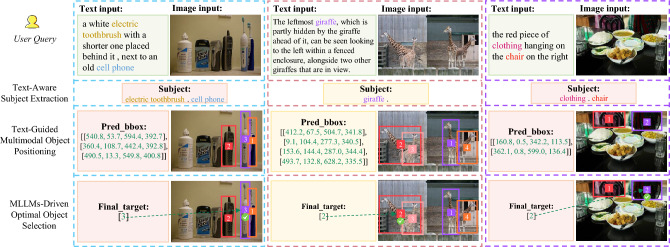
Representative examples illustrating the proposed MQADet paradigm. Each case shows the specific input to MQADet and the corresponding outputs across its three stages. The results demonstrate that MQADet can accurately reason over complex queries and effectively identify a broader range of object categories.

Given an image *I* and a complex textual query *T*, the TASE stage identifies the target subjects described in the query along with their corresponding semantic features. The TMOP stage subsequently employs a state-of-the-art object detector to generate candidate bounding boxes and assign numerical marks corresponding to the identified subjects. Finally, the MOOS stage bridges the gap between perception and reasoning, producing the optimal detection result through the MQA mechanism. The following sections provide detailed explanations of each stage. Figure 3 presents representative examples illustrating the proposed MQADet paradigm.

### Text-aware subject extraction (TASE)

Open-vocabulary detection in real-world scenarios is inherently complex, requiring the coordination of multiple subtasks to achieve robust performance. For instance, users often aim to detect specific targets described with detailed sentences, such as “construction worker with a yellow helmet, reflective safety jacket, and pants”, rather than simple targets lacking descriptive features like “guy”, “car”, or “banana”. However, most existing OVD models exhibit limited capability in handling such complex linguistic descriptions. Following the principle of multimodal decomposition^23^, we adopt a strategy that decomposes complex tasks into a sequence of simpler subtasks. This decomposition forms the foundation of the MQADet paradigm for effectively tackling the challenges of textual complexity.

At the core of MQADet lies the integration of multimodal large language models (MLLMs), which have demonstrated remarkable zero-shot and few-shot reasoning performance. To overcome the limitations of existing OVD models, we introduce the Text-Aware Subject Extraction (TASE) stage as the first phase of MQADet. This stage leverages MLLMs and common-sense knowledge to parse and identify the target subjects, denoted as $\{OS_i\}_{i=1}^{M}$, from the input query *T*, which can be formulated as:

1 $$\begin{aligned} \{OS_i\}_{i=1}^{M} = MLLMs (T) \end{aligned}$$

where *M* denotes the number of target subjects in the query. These subjects $\{OS_i\}$, representing the entities and their descriptive features, are passed to the next stage of MQADet. The details of the prompts utilized for the MLLMs in this stage are provided in Section 5.3.1.

### Text-guided multimodal object positioning (TMOP)

In the previous stage, the target subjects and their corresponding semantic features are extracted from the user query text. The TMOP stage then processes these extracted subjects along with the input image *I* to generate candidate bounding boxes and assign corresponding object marks.

Specifically, a state-of-the-art open-vocabulary detector (e.g., Grounding DINO, YOLO-World, or OmDet-Turbo) is incorporated into this stage to automatically identify potential object regions. For each identified subject $\{OS_i\}$, the detector produces a set of candidate bounding boxes $\{Boxes_i\}$ and assigns a unique object mark $\{Marks_{ij}\}_{j=1}^{P}$ to each region, where *P* denotes the number of candidate regions detected for the *i*-th subject and *j* indexes the *j*-th candidate. Each $\{Marks_{ij}\}$ is a numeric identifier (i.e., an index) visually rendered at the center of its corresponding bounding box, providing an explicit reference for later multimodal reasoning. Importantly, these marks are perceived directly through visual recognition by MLLMs (GPT-4o and LLaVA-1.5) from the marked image, without supplying any external textual list, coordinate information, or other structured annotations. This process can be mathematically expressed as:

2 $$\begin{aligned} \{Boxes_i, Marks_i\} = Dets (OS_i, I) \end{aligned}$$

3 $$\begin{aligned} MI = I (Boxes, Marks) \end{aligned}$$

Here, *Dets* denotes the selected detector, *i* corresponds to the *i*-th object subject, *I* is the original query image, and *MI* represents the resulting marked image containing the detected boxes and their respective indices.

A key advantage of MQADet is its flexibility: the TMOP stage enables seamless integration of various state-of-the-art detectors without requiring additional fine-tuning or costly training. This design accommodates the rapid evolution of modern vision-language models and allows efficient utilization of existing detection architectures. The resulting marked image (*MI*) is then passed to the final stage, where the MLLMs-driven Optimal Object Selection (MOOS) module further refines and verifies the detection results.

### MLLMs-driven optimal object selection (MOOS)

The final stage of MQADet, termed MLLMs-Driven Optimal Object Selection (MOOS), ensures fine-grained alignment between visual targets and complex linguistic descriptions, ultimately producing the optimal detection result *OB*. This stage reformulates the final detection step as a choice-based Multimodal Question Answering (MQA) task, enabling the MLLMs to reason over both textual semantics and the visually annotated candidate regions.

Given the marked image *MI*–which contains all candidate bounding boxes visually indexed with numeric identifiers–and the corresponding query text *T*, the MLLMs are prompted to determine which candidate region best matches the description. This process is formulated as:

4 $$\begin{aligned} OB = MLLMs(T, MI) \end{aligned}$$

Modern MLLMs (e.g., GPT-4o and LLaVA) have demonstrated strong capabilities in interpreting visual content and aligning it with complex textual descriptions, as reported in their official evaluations. MOOS leverages these capabilities by providing explicit, visually indexed candidate regions in *MI*, allowing the MLLMs to directly compare the visual evidence with the compositional semantics in *T*. This explicit grounding ensures that the final selection is guided by observable visual-semantic consistency rather than coarse category associations. The effectiveness of this mechanism is further supported by our ablation studies, where removing MOOS results in a substantial performance drop, underscoring its essential role in accurate object selection.

In this work, GPT-4o and LLaVA are employed as the MLLMs for the MOOS stage. The detailed design of the instruction prompts used in this process is provided in Section 5.3.3.

All methods were carried out in accordance with relevant guidelines and regulations, and no ethical approval or informed consent is required as only publicly available datasets are used.

## Benchmark

### Datasets and evaluation metrics

#### Datasets

To comprehensively evaluate the zero-shot detection capability of MQADet under open-vocabulary (OV) settings, we conduct experiments on four widely adopted benchmark datasets: RefCOCO, RefCOCO+, RefCOCOg, and Ref-L4. These datasets are characterized by rich and complex textual descriptions, providing diverse scenarios for assessing the vision-language alignment and reasoning ability of object detectors.

**RefCOCO**^24^, **RefCOCO+**^24^, and **RefCOCOg**^25^ are benchmark datasets for referring expression comprehension, where natural language expressions are used to localize specific objects within images. Among them, RefCOCO+ excludes spatial prepositions such as “on the right”, focusing on appearance-based reasoning, whereas RefCOCOg incorporates spatial relations and includes longer, more descriptive expressions. The average query lengths are 3.61, 3.53, and 8.43 words for RefCOCO, RefCOCO+, and RefCOCOg, respectively, reflecting their progressive increase in linguistic complexity.

**Ref-L4**^26^ is a recently introduced large-scale benchmark for open-vocabulary object detection. It contains 365 distinct object categories with instance counts ranging from 30 to 3,767. Notably, Ref-L4 features lengthy referring expressions averaging 24.2 words and an extensive vocabulary of 22,813 unique words, making it a particularly challenging dataset for evaluating fine-grained visual-textual reasoning and generalization.

The RefCOCO, RefCOCO+, and RefCOCOg datasets are publicly available at: https://github.com/shikras/shikra. The Ref-L4 dataset is publicly available at: https://github.com/JierunChen/Ref-L4.

#### Evaluation metrics

To quantitatively evaluate the detection performance of different models, we adopt three widely used metrics: Acc@0.25, Acc@0.5, and $\Delta$, following prior works^27–30^. Specifically, Acc@0.25 and Acc@0.5 measure the accuracy of bounding box predictions, where a prediction is considered correct if the Intersection-over-Union (IoU) between the predicted bounding box and the ground-truth box exceeds thresholds of 0.25 and 0.5, respectively. The metric $\Delta$ denotes the relative improvement of MQADet compared with the baseline models.

The IoU and accuracy metrics are formally defined as:

5 $$\begin{aligned} IoU = \frac{area(B_p \cap B_{gt})}{area(B_p \cup B_{gt})} \end{aligned}$$

6 $$\begin{aligned} Acc@IoU(T) = \frac{1}{N}\sum \limits _{i=1}^{N} 1(IoU_i \ge T) \end{aligned}$$

where $B_p$ and $B_{gt}$ denote the predicted and ground-truth bounding boxes, respectively. The IoU measures the overlap ratio between these two boxes, with higher values indicating greater localization accuracy. *N* represents the total number of ground-truth instances, and $\textbf{1}(IoU_i \ge T)$ is an indicator function that equals 1 if the *i*-th prediction satisfies the IoU threshold *T*, and 0 otherwise. In this study, *T* is set to 0.25 and 0.5, corresponding to the Acc@0.25 and Acc@0.5 metrics.

### Baselines

For fair and comprehensive evaluation, we compare MQADet against three state-of-the-art open-vocabulary (OV) object detectors–Grounding DINO, YOLO-World, and OmDet-Turbo–and employ two representative multimodal large language models (MLLMs), GPT-4o and LLaVA-1.5, as reasoning backbones.

#### Detector baselines

**Grounding DINO.** Grounding DINO^10^ is a powerful open-set object detector capable of identifying arbitrary objects based on human-provided textual inputs such as category names or referring expressions. It extends traditional closed-set detectors by incorporating a text encoder, enabling robust open-vocabulary detection with strong zero-shot generalization.

**YOLO-World.** YOLO-World^11^ is a cutting-edge zero-shot object detection framework that unifies visual and textual representations for OV detection. Unlike conventional YOLO architectures, it integrates a pre-trained CLIP text encoder to support text-based object recognition without additional fine-tuning. The model maintains the lightweight efficiency and rapid inference speed of the YOLO family, making it practical for real-time deployment.

**OmDet-Turbo.** OmDet-Turbo^12^ is a transformer-based open-vocabulary detector optimized for real-time performance. It achieves a strong balance between accuracy and efficiency, demonstrating superior detection quality and inference speed in diverse zero-shot detection scenarios.

#### MLLM baselines

**GPT-4o.** GPT-4o (https://openai.com/index/hello-gpt-4o/.) is a multimodal large language model capable of processing and reasoning over text, image, and audio inputs simultaneously. Its advantages include: (1) real-time interaction with minimal latency, (2) response generation twice as fast as GPT-4 Turbo (https://help.openai.com/en/articles/8555510-gpt-4-turbo.), and (3) strong cross-modal reasoning and visual grounding capabilities, making it particularly suitable for MQA-based detection tasks.

**LLaVA-1.5.** The Large Language and Vision Assistant (LLaVA)^31^ is an end-to-end multimodal model that connects a vision encoder with a large language model (LLM) for unified vision-language understanding. LLaVA-1.5^32^ enhances the original LLaVA architecture by incorporating a CLIP-ViT-L/336px visual encoder with an MLP projection and introducing academically curated visual question answering (VQA) data along with structured instruction prompts, thereby improving its reasoning performance in open-domain visual tasks.

## Experiments

### Implementation details

In our experiments, we employ two multimodal large language models (MLLMs), *gpt-4o* and *llava-v1.5-7b*, as the reasoning modules within the MQADet paradigm. During the TMOP stage, three state-of-the-art open-vocabulary object detectors–Grounding DINO, YOLO-World, and OmDet-Turbo–are utilized as visual backbones. Specifically, for Grounding DINO, we set the *box_threshold* and *text_threshold* to 0.25 and adopt *GroundingDINO-T* as the inference model; as it does not provide a parameter for the number of candidate boxes, the default setting is used. For YOLO-World, we employ *YOLO-Worldv2-XL* with default *topk = 100* candidate boxes and a confidence threshold of 0.30. For OmDet-Turbo, the inference model is *OmDet-Turbo_tiny_SWIN_T*; as it also lacks a parameter for the number of candidate boxes, the default is used, with *conf_threshold = 0.30* and *nms_threshold = 0.5*. The checkpoints used for all detectors and MLLMs are summarized in Section 5.2.

For evaluation, we conduct experiments on four benchmark datasets: RefCOCO, RefCOCO+, RefCOCOg, and Ref-L4. To ensure balanced and computationally efficient evaluation, we uniformly sample 10% of the data from each dataset. After sampling, RefCOCO contains 12,062 expressions in the training set, 565 in testA, 509 in testB, and 1,083 in the validation set. RefCOCO+ includes 12,019 expressions in the training set, 572 in testA, 488 in testB, and 1,075 in the validation set. RefCOCOg consists of 8,051 expressions in the training set, 960 in the test set, and 489 in the validation set. Ref-L4 comprises 3,192 expressions in the test set and 1,342 in the validation set.

All experiments are conducted on a single NVIDIA RTX 4090 GPU. The detailed processing pipeline, including the input and output results of each MQADet stage, is described in Section 5.3.

### Model details

The specific models and their corresponding checkpoints used in the MQADet paradigm are summarized in Table 1. All open-source models were directly obtained from publicly available repositories on Hugging Face or GitHub. The selected pretrained weights for each model are listed below.

**Table 1:** Checkpoints of the models employed in MQADet.

**Model**	**Checkpoints**
Grounding DINO	groundingdino_swint_ogc.pth
YOLO-World	yolo_world_v2_xl_obj365v1_goldg_cc3mlite_pretrain.pth
OmDet-Turbo	OmDet-Turbo_tiny_SWIN_T.pth
LLaVA-1.5	liuhaotian/llava-v1.5-7b

Since GPT-4o is not open-sourced, its internal checkpoints are not publicly accessible. Therefore, all evaluations involving GPT-4o were conducted using the official API provided by OpenAI across the benchmark datasets.

### MQADet details

To ensure consistency and reproducibility of the experimental results, identical prompts were employed for the MLLMs (GPT-4o and LLaVA) in both the TASE and MOOS stages of MQADet. The following subsections detail the prompt design, as well as the corresponding inputs and outputs for each stage of MQADet, illustrated through a representative example.

**Fig. 4:**
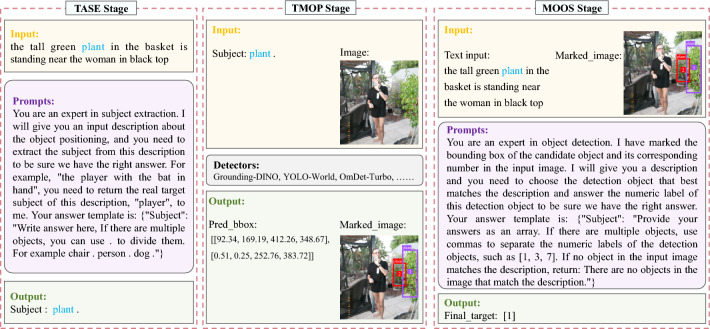
Illustration of the specific prompts and detectors, along with corresponding inputs and outputs, across the TASE, TMOP, and MOOS stages of the proposed MQADet paradigm.

#### TASE stage details

The Text-Aware Subject Extraction (TASE) stage takes as input a complex textual query describing the target object(s).

- **Input**: The original complex text input from the user query. Example input:
  - *”Text input”: “the tall green plant in the basket is standing near the woman in black top”*

- **Prompts**: In this stage, we explicitly guide the MLLMs (GPT-4o and LLaVA-1.5) to output a structured list of subjects. The models are prompted using the following instruction (also shown in Figure 4):
  - *You are an expert in subject extraction. I will give you an input description about the object positioning, and you need to extract the subject from this description to be sure we have the right answer. For example, “the player with the bat in hand”, you need to return the real target subject of this description, “player”, to me. Your answer template is: {“Subject”: “Write answer here, If there are multiple objects, you can use. to divide them. For example chair. person. dog.” } *

- **Output**: The MLLMs produce a formatted subject list strictly following the predefined template. The output string is then parsed to construct the final subject set $\{OS_i\}$ used in subsequent stages of our paradigm. When multiple subjects are extracted, MQADet preserves their order in the query, and this order defines the priority for generating candidate bounding boxes in the TMOP stage. Example output:
  - *“Subject”: “plant.”*

#### TMOP stage details

The Text-Guided Multimodal Object Positioning (TMOP) stage generates a set of candidate bounding boxes, assigns a numeric mark to each box, and produces the corresponding marked image, as illustrated in Figure 4.

- **Input**: The subject prompts derived from TASE and the original image. Example input:
  - *“Subject”: “plant.”*
  - *the original image*
- **Output**: The candidate bounding box coordinates ([x_min, y_min, x_max, y_max]), their associated numeric marks, and the resulting marked image. Example output:
  - *“Pred_bbox”: [[92.34, 169.19, 412.26, 348.67], [0.51, 0.25, 252.76, 383.72]]*
  - *the marked image*

This stage adopts a fully plug-and-play design: any state-of-the-art open-vocabulary detector can be integrated without additional training or fine-tuning. This flexibility allows MQADet to leverage the rapid evolution of modern detection models while ensuring efficient localization of diverse candidate targets under OV settings.

#### MOOS stage details

The MLLMs-Driven Optimal Object Selection (MOOS) stage performs fine-grained reasoning to align the original textual description with the visual candidates obtained from TMOP.

- **Input**:
  - *“Text input”: “the tall green plant in the basket is standing near the woman in black top”*
  - *the marked image*

- **Prompts**: In this stage, carefully designed prompts (Figure 4) guide the MLLMs (GPT-4o and LLaVA-1.5) to reason over the candidate regions and identify the optimal match to the query. The following instruction prompts are used:
  - *You are an expert in object detection. I have marked the bounding box of the candidate object and its corresponding number in the input image. I will give you a description and you need to choose the detection object that best matches the description and answer the numeric label of this detection object to be sure we have the right answer. Your answer template is: “Subject”: “Provide your answers as an array. If there are multiple objects, use commas to separate the numeric labels of the detection objects, such as [1, 3, 7]. If no object in the input image matches the description, return: There are no objects in the image that match the description.” *

- **Output**: The final target(s) selected through MLLMs-based multimodal reasoning. Example output:
  - *“Final_target”: [1]* (where [1] denotes the index of the correctly matched object)

### Main results

#### Performance on GPT-4o

Table 2 reports the results of MQADet with GPT-4o on four distinct benchmarks (RefCOCO, RefCOCO+, RefCOCOg, and Ref-L4), comparing it with three representative open-vocabulary detectors: Grounding DINO, YOLO-World, and OmDet-Turbo. Across all datasets and metrics, MQADet consistently and significantly improves every detector baseline under the same experimental settings.

**Table 2:** Results comparison between MQADet and state-of-the-art detectors on RefCOCO/+/g, and Ref-L4. The MLLM employs GPT-4o, while object detectors utilize Grounding DINO^10^, YOLO-World^11^, and OmDet-Turbo^12^. Evaluation metrics include Acc@0.5, Acc@0.25, and $\Delta$. Values in italic indicate improvement gains over the detector baselines.

**Method**	**Metric**	**RefCOCO** ^24^				**RefCOCO+** ^24^				**RefCOCOg** ^25^			**Ref-L4** ^26^	
		train	val	testA	testB	train	val	testA	testB	train	val	test	val	test
G-DINO	Acc@0.25	48.00	48.95	49.83	40.50	48.14	49.66	50.58	43.51	42.21	40.76	41.96	17.40	17.19
	Acc@0.5	43.14	42.85	45.07	36.69	41.77	41.56	43.98	37.51	39.43	38.18	39.24	16.66	16.34
MQADet + G-DINO	Acc@0.25	64.70	66.59	64.01	67.20	57.35	57.29	55.07	56.87	66.52	66.10	67.91	63.71	64.21
	$\Delta$	*+16.7*	*+17.64*	*+14.18*	*+26.7*	*+9.21*	*+7.63*	*+4.49*	*+13.36*	*+24.31*	*+25.34*	*+25.95*	*+46.31*	*+47.02*
	Acc@0.5	58.92	60.47	60.03	61.70	50.62	49.50	48.51	50.18	61.58	61.45	62.90	59.35	59.34
	$\Delta$	*+15.78*	*+17.62*	*+14.96*	*+25.01*	*+8.85*	*+7.94*	*+4.53*	*+12.67*	*+22.15*	*+23.27*	*+23.66*	*+42.69*	*+43.0*
YOLO-World	Acc@0.25	38.79	38.15	42.70	32.97	39.24	37.82	38.20	35.32	42.44	40.11	43.05	28.76	29.94
	Acc@0.5	34.09	32.65	38.36	28.47	33.56	31.06	33.77	30.65	38.43	36.99	38.51	25.25	26.56
MQADet + YOLO-World	Acc@0.25	63.72	62.79	60.59	62.13	56.15	56.97	52.91	55.47	66.15	62.50	65.57	62.98	57.75
	$\Delta$	*+24.93*	*+24.64*	*+17.89*	*+29.16*	*+16.91*	*+19.15*	*+14.71*	*+20.15*	*+23.71*	*+22.39*	*+22.52*	*+34.22*	*+27.81*
	Acc@0.5	57.98	56.81	55.28	55.65	49.76	48.31	46.88	48.84	61.17	57.55	60.44	57.86	53.22
	$\Delta$	*+23.89*	*+24.16*	*+16.92*	*+27.18*	*+16.2*	*+17.25*	*+13.11*	*+18.19*	*+22.74*	*+20.56*	*+21.93*	*+32.61*	*+26.66*
OmDet-Turbo	Acc@0.25	49.62	48.87	55.44	41.38	48.07	46.84	49.03	44.09	46.02	42.81	45.01	32.29	32.16
	Acc@0.5	46.53	45.43	52.76	37.06	44.57	42.96	46.03	37.94	40.79	38.27	39.07	28.67	28.95
MQADet + OmDet-Turbo	Acc@0.25	62.04	58.34	64.48	50.56	54.59	54.34	55.91	51.59	59.20	56.82	57.62	56.94	54.06
	$\Delta$	*+12.42*	*+9.47*	*+9.04*	*+9.18*	*+6.52*	*+7.5*	*+6.88*	*+7.5*	*+13.18*	*+14.01*	*+12.61*	*+24.65*	*+21.9*
	Acc@0.5	58.07	53.77	61.39	45.84	50.07	49.46	53.21	46.47	54.55	52.22	52.90	51.66	49.55
	$\Delta$	*+11.54*	*+8.34*	*+8.63*	*+8.78*	*+5.5*	*+6.5*	*+7.18*	*+8.53*	*+13.76*	*+13.95*	*+13.83*	*+22.99*	*+20.6*

Specifically, MQADet brings large performance gains for both Acc@0.25 and Acc@0.5. At the stricter Acc@0.5 threshold, MQADet improves Grounding DINO, YOLO-World, and OmDet-Turbo by up to 43.0%, 26.66%, and 20.6%, respectively. Similar improvements are observed for Acc@0.25, where the gains reach 47.02%, 27.81%, and 21.9%. These results further highlight MQADet’s strong generality and its ability to enhance detectors of very different architectures.

**Fig. 5:**
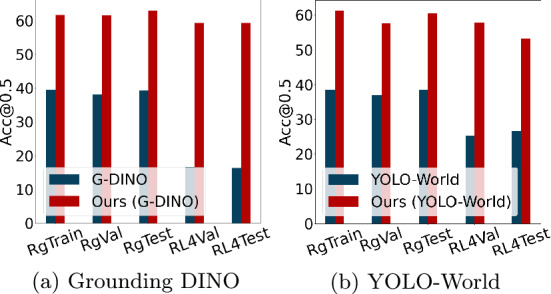
Performance comparison between MQADet and baseline detectors on challenging RefCOCOg and Ref-L4 datasets. The MLLM employs GPT-4o, while object detectors utilize Grounding DINO and YOLO-World. The evaluation metric is Acc@0.5. RgTrain, RgVal, RgTest, RL4Val, and RL4Test denote the RefCOCOg train/val/test and Ref-L4 val/test sets, respectively.

Figure 5 further illustrates that MQADet maintains substantial and stable advantages on the more challenging RefCOCOg and Ref-L4 benchmarks, both of which contain long, compositional, and linguistically complex queries. The unified three-stage design of MQADet–comprising subject extraction, detector-guided grounding, and reasoning-based object selection–enables explicit handling of fine-grained semantics and complex visual-textual correspondence. With GPT-4o’s cross-modal reasoning, MQADet effectively corrects detector misalignment and significantly improves open-vocabulary grounding performance.

#### Performance on LLaVA-1.5

To further evaluate the robustness and transferability of the proposed paradigm, we replace GPT-4o with LLaVA-1.5 and report the results in Table 3. MQADet continues to deliver consistent and noticeable improvements across all benchmarks and detectors, demonstrating that the effectiveness of the paradigm does not depend on a specific MLLM.

Using LLaVA-1.5, MQADet still achieves the best performance on all datasets for both Acc@0.25 and Acc@0.5, and the $\Delta$ scores show clear improvement over the detector baselines. Notably, even when switching to a lighter open-source MLLM, MQADet provides substantial enhancements for Grounding DINO, YOLO-World, and OmDet-Turbo, confirming the paradigm’s broad applicability.

These results demonstrate that MQADet is MLLM-agnostic and detector-agnostic: its three-stage MQA framework reliably improves visual-textual alignment, enables more accurate grounding of complex descriptions, and strengthens open-vocabulary detection performance without requiring any retraining or architectural modification. This plug-and-play property also underscores MQADet’s practicality and scalability for real-world applications.

**Table 3:** Results comparison between MQADet and state-of-the-art detectors on RefCOCO/+/g, and Ref-L4. The MLLM employs LLaVA-1.5^33^, while object detectors utilize Grounding DINO^10^, YOLO-World^11^, and OmDet-Turbo^12^. Evaluation metrics include Acc@0.5, Acc@0.25, and $\Delta$. Values in italic indicate improvement gains over the detector baselines.

**Method**	**Metric**	**RefCOCO**^24^				**RefCOCO+**^24^				**RefCOCOg**^25^			**Ref-L4**^26^	
		train	val	testA	testB	train	val	testA	testB	train	val	test	val	test
G-DINO	Acc@0.25	48.00	48.95	49.83	40.50	48.14	49.66	50.58	43.51	42.21	40.76	41.96	17.40	17.19
	Acc@0.5	43.14	42.85	45.07	36.69	41.77	41.56	43.98	37.51	39.43	38.18	39.24	16.66	16.34
MQADet + G-DINO	Acc@0.25	58.75	53.92	56.46	52.26	55.16	53.58	56.12	51.64	67.54	66.05	67.40	54.40	45.21
	$\Delta$	*+10.75*	*+4.97*	*+6.63*	*+11.76*	*+7.02*	*+3.92*	*+5.54*	*+8.13*	*+25.33*	*+25.29*	*+25.44*	*+37.0*	*+28.02*
	Acc@0.5	51.79	46.45	51.33	45.19	47.16	44.09	49.13	43.03	61.14	59.51	61.15	49.85	41.13
	$\Delta$	*+8.65*	*+3.6*	*+6.26*	*+8.5*	*+5.39*	*+2.53*	*+5.15*	*+5.52*	*+21.71*	*+21.33*	*+21.91*	*+33.19*	*+24.79*
YOLO-World	Acc@0.25	38.79	38.15	42.70	32.97	39.24	37.82	38.20	35.32	42.44	40.11	43.05	28.76	29.94
	Acc@0.5	34.09	32.65	38.36	28.47	33.56	31.06	33.77	30.65	38.43	36.99	38.51	25.25	26.56
MQADet + YOLO-World	Acc@0.25	55.66	46.63	45.84	43.22	54.12	51.72	50.52	45.29	63.01	50.31	64.38	53.13	34.77
	$\Delta$	*+16.87*	*+8.48*	*+3.14*	*+10.25*	*+14.88*	*+13.9*	*+12.32*	*+9.97*	*+20.57*	*+10.2*	*+21.33*	*+24.37*	*+4.83*
	Acc@0.5	48.97	39.34	40.71	36.74	45.87	42.60	44.76	36.07	56.49	44.99	56.88	48.14	31.42
	$\Delta$	*+14.88*	*+6.69*	*+2.35*	*+8.27*	*+12.31*	*+11.54*	*+10.99*	*+5.42*	*+18.06*	*+8.0*	*+18.37*	*+22.89*	*+4.86*
OmDet-Turbo	Acc@0.25	49.62	48.87	55.44	41.38	48.07	46.84	49.03	44.09	46.02	42.81	45.01	32.29	32.16
	Acc@0.5	46.53	45.43	52.76	37.06	44.57	42.96	46.03	37.94	40.79	38.27	39.07	28.67	28.95
MQADet + OmDet-Turbo	Acc@0.25	59.89	58.17	62.83	50.88	56.04	54.05	54.20	47.95	63.05	66.67	71.88	52.83	48.68
	$\Delta$	*+10.27*	*+9.3*	*+7.39*	*+9.5*	*+7.97*	*+7.21*	*+5.17*	*+3.86*	*+17.03*	*+23.86*	*+26.87*	*+20.54*	*+16.52*
	Acc@0.5	53.65	50.78	57.52	43.03	49.54	47.81	50.87	41.60	56.35	60.12	62.60	47.47	42.95
	$\Delta$	*+7.12*	*+5.35*	*+4.76*	*+5.97*	*+4.97*	*+4.85*	*+4.84*	*+3.66*	*+15.56*	*+21.85*	*+23.53*	*+18.8*	*+14.0*

#### Comparison with the state-of-the-arts

We conduct comprehensive comparisons between MQADet and representative vision-language models (VLMs), including DeepSeek-VL2-Tiny, Qwen2-VL-2B, and the stronger MLLM Gemini-2.0-Flash-Lite. The results on the RefCOCO testA and Ref-L4 val datasets are shown in Table 4.

DeepSeek-VL2-Tiny and Qwen2-VL-2B perform well on the RefCOCO testA, where referring expressions are relatively short and simple. However, their accuracy decreases markedly on the Ref-L4 dataset, which contains longer and more complex descriptions. This indicates that lightweight VLMs face limitations in handling compositional semantics and long-range linguistic dependencies. To provide a more balanced evaluation across models with different capacities, we further include Gemini-2.0-Flash-Lite, a more capable MLLM with visual grounding abilities. Its improved performance on Ref-L4 highlights the importance of a stronger reasoning capability when dealing with complex linguistic queries.

Across all detector choices, MQADet maintains stable performance on both datasets. Although its accuracy on the RefCOCO testA is slightly affected by the reliance on detected proposals, MQADet achieves competitive or superior accuracy on Ref-L4. The three-stage design–text-aware subject extraction, text-guided multimodal object positioning, and MLLMs-driven optimal object selection–enables effective handling of long and complex referring expressions.

Overall, MQADet performs reliably across simple and complex benchmarks and competes effectively with both lightweight end-to-end VLMs and stronger MLLMs, demonstrating the robustness of the proposed paradigm.

**Table 4:** Comparison with state-of-the-art models on the RefCOCO testA and Ref-L4 val datasets. MQADet employs GPT-4o as the MLLM, while object detectors utilize Grounding DINO^10^, YOLO-World^11^, and OmDet-Turbo^12^.

Method	RefCOCO testA		Ref-L4 val	
	Acc@0.25	Acc@0.5	Acc@0.25	Acc@0.5
DeepSeek-VL2-Tiny^34^	83.72	80.35	4.84	3.20
Qwen2-VL-2B^35^	82.12	76.99	24.22	18.55
Gemini-2.0-Flash-Lite^36^	73.86	59.87	59.87	46.01
MQADet + G-DINO	64.01	60.03	63.71	59.35
MQADet + YOLO-World	60.59	55.28	62.98	57.86
MQADet + OmDet-Turbo	64.48	61.39	56.94	51.66

### Ablation experiments

To validate the contribution of each stage in MQADet’s three-stage multimodal reasoning pipeline, we conducted ablation studies on the RefCOCO testA and Ref-L4 val datasets. In each variant, one or more stages were removed to assess their individual impact on performance. GPT-4o was used as the MLLM, and Grounding DINO, YOLO-World, and OmDet-Turbo served as detectors in the TMOP stage.

As shown in Table 5, the complete MQADet configuration achieves the best overall performance on both datasets. Removing the TASE stage (text-aware subject extraction) or the MOOS stage (MLLMs-driven optimal object selection) leads to a substantial decline in detection accuracy. This highlights the importance of both linguistic parsing for accurate subject identification and cross-modal reasoning for fine-grained visual-textual alignment.

These findings confirm the effectiveness of MQADet’s three-stage design in enhancing the reasoning ability of open-vocabulary detectors under diverse and linguistically complex scenarios.

**Fig. 6:**
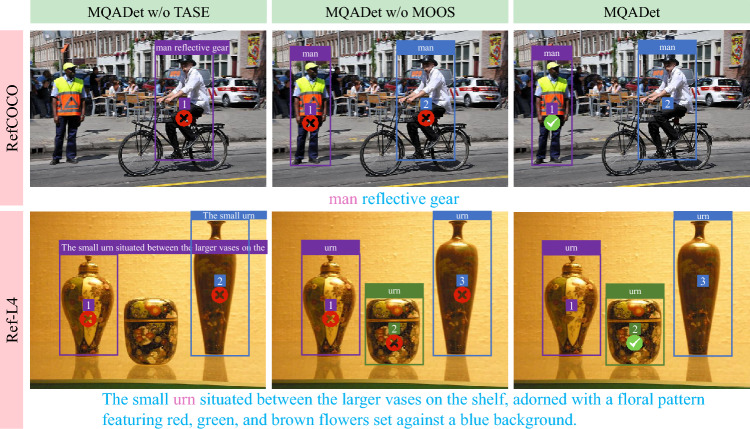
Visual comparison of complete MQADet, without TASE, and without MOOS on samples from RefCOCO and Ref-L4. Pink words denote the subjects identified from the user query.

Figure 6 presents representative cases from RefCOCO and Ref-L4, showing that removing TASE causes incorrect subject interpretation and removing MOOS leads to suboptimal region selection, whereas the complete MQADet consistently achieves accurate grounding.

**Table 5:** Ablation study on RefCOCO testA and Ref-L4 val datasets. GPT-4o is used as the MLLM, and object detectors (Grounding DINO^10^, YOLO-World^11^, and OmDet-Turbo^12^) are employed in the TMOP stage.

TASE Stage	TMOP Stage			MOOS Stage	RefCOCO testA		Ref-L4 val	
	G-DINO	YOLO-World	OmDet-Turbo		Acc@0.25	Acc@0.5	Acc@0.25	Acc@0.5
	$\checkmark$			$\checkmark$	44.68	40.78	54.97	44.76
		$\checkmark$		$\checkmark$	54.69	48.85	36.74	43.04
			$\checkmark$	$\checkmark$	56.31	54.90	49.96	43.07
$\checkmark$	$\checkmark$				43.80	40.42	37.82	36.28
$\checkmark$		$\checkmark$			43.79	39.67	40.15	37.65
$\checkmark$			$\checkmark$		50.15	47.00	44.52	40.87
$\checkmark$	$\checkmark$			$\checkmark$	**64.01**	**60.03**	**63.71**	**59.35**
$\checkmark$		$\checkmark$		$\checkmark$	**60.59**	**55.28**	**62.98**	**57.86**
$\checkmark$			$\checkmark$	$\checkmark$	**64.48**	**61.39**	**56.94**	**51.66**

The inference-time analysis in Table 6 further illustrates the efficiency of the proposed three-stage paradigm. Although MQADet adopts a multi-step pipeline, its overall latency remains competitive. Using LLaVA-1.5 as the MLLM and YOLO-World as the detector, MQADet achieves a total inference time of 1000.9 ms, which is substantially faster than the lightweight end-to-end model Qwen2-VL-2B (1951.7 ms) and far below the larger MLLM Gemini-2.0-Flash-Lite (7364.9 ms). The TASE and TMOP stages introduce only minor overheads of 87.6 ms and 34.7 ms, respectively, while the MOOS stage accounts for most of the latency due to its fine-grained multimodal reasoning. Despite this additional reasoning step, MQADet preserves a favorable balance between accuracy and computational cost, demonstrating that the proposed three-stage framework achieves efficient inference while enabling more reliable alignment between textual descriptions and visual regions.

**Table 6:** Analysis of time consumption for Qwen2-VL, Gemini-2.0 and MQADet, where MQADet comprises three stages. MQADet employs LLaVA-1.5^33^ as the MLLM, while the object detector utilizes YOLO-World^11^.

Method	Stages	Inference Time (ms)
Qwen2-VL-2B^35^	$\setminus$	1951.7
Gemini-2.0-Flash-Lite^36^	$\setminus$	7364.9
MQADet + YOLO-World	TASE Stage	87.6
	TMOP Satge	34.7
	MOOS Satge	878.6
	Total	1000.9

**Fig. 7:**
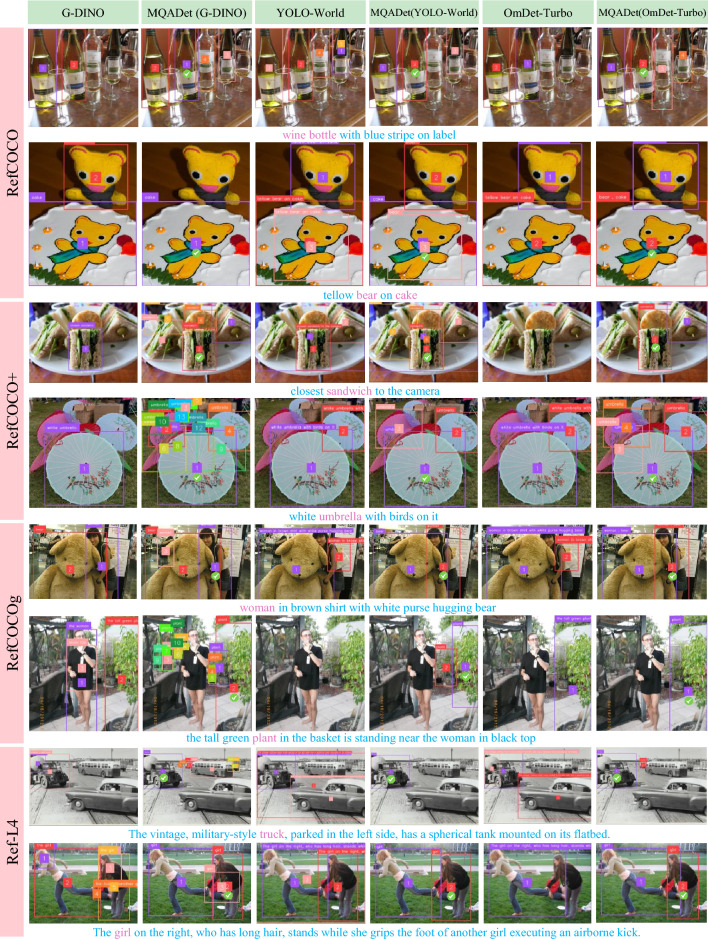
Qualitative comparison between MQADet and three state-of-the-art open-vocabulary (OV) detectors–Grounding DINO, YOLO-World, and OmDet-Turbo–on the RefCOCO/+/g, and Ref-L4 datasets, with GPT-4o employed as the MLLM. Pink words denote the subjects identified from the user query.

### Visualizations

Our proposed MQADet paradigm significantly enhances the capabilities of existing state-of-the-art open-vocabulary detectors. To provide intuitive evidence, we present visualization results using both GPT-4o and LLaVA-1.5 as the employed MLLMs.

#### Visualization on GPT-4o

Figure 7 presents a qualitative comparison between MQADet and three leading OV detectors–Grounding DINO, YOLO-World, and OmDet-Turbo–on four benchmark datasets (RefCOCO, RefCOCO+, RefCOCOg, and Ref-L4), with GPT-4o serving as the MLLM. Specifically, the first, third, and fifth columns correspond to predictions from Grounding DINO, YOLO-World, and OmDet-Turbo, respectively, while the remaining columns illustrate results from our MQADet paradigm.

The visualization results clearly demonstrate that MQADet enables detectors to attend to a broader range of object categories by leveraging subject cues extracted in the TASE stage. Furthermore, the integration of MLLMs-driven reasoning enhances the fine-grained alignment between visual and textual information. Overall, these results highlight the robust zero-shot detection capability and strong cross-modal reasoning ability of MQADet across all benchmark datasets.

#### Visualization on LLaVA-1.5

We further analyze visualization results with LLaVA-1.5 employed as the MLLM across the same set of detectors and datasets to assess the paradigm’s transferability. The qualitative comparisons indicate that, regardless of whether GPT-4o or LLaVA-1.5 is adopted as the reasoning model, MQADet effectively bridges the gap between perception and reasoning. It successfully mitigates challenges caused by complex visual-textual misalignment and substantially improves detection accuracy in open-vocabulary scenarios.

**Fig. 8:**
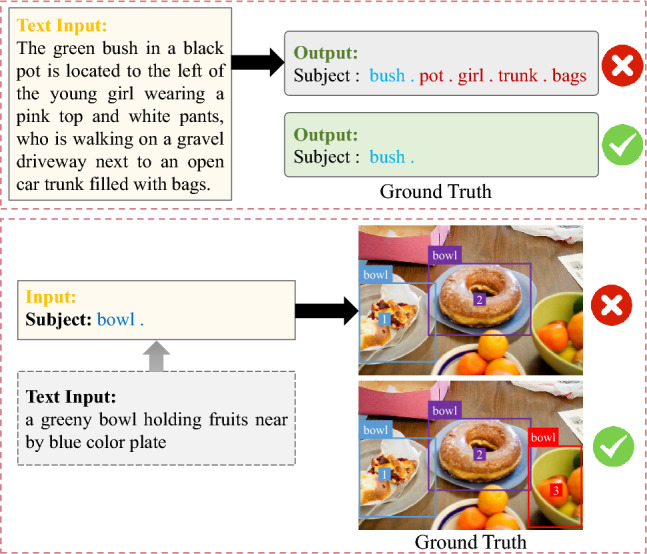
Failure cases of MQADet: (a) incorrect subject extraction from a complex query; (b) missed detection of small or occluded targets.

## Limitations

MQADet may still exhibit failures due to limitations of current MLLMs and open-vocabulary detectors rather than the three-stage paradigm itself. Complex queries can cause inaccurate subject extraction in TASE, while small, occluded, or ambiguous objects may be missed during TMOP, leading to errors (Figure 8). These limitations are expected to decrease as MLLMs and detectors advance, highlighting opportunities for further improvement.

## Conclusion

Existing open-vocabulary object detectors often struggle with complex textual queries and fine-grained misalignment between visual regions and linguistic descriptions. To address these challenges, we proposed MQADet, a three-stage multimodal reasoning paradigm comprising Text-Aware Subject Extraction (TASE), Text-Guided Multimodal Object Positioning (TMOP), and MLLMs-Driven Optimal Object Selection (MOOS). By combining the perceptual capabilities of open-vocabulary detectors with the reasoning power of MLLMs, MQADet substantially enhances the OV detection performance of existing detectors, enabling accurate and interpretable object grounding in challenging visual-textual scenarios. Extensive experiments on RefCOCO, RefCOCO+, RefCOCOg, and Ref-L4 demonstrate that MQADet consistently outperforms state-of-the-art methods, highlighting its robustness, generalizability, and potential for advancing real-world open-vocabulary detection.

## Data Availability

The datasets used and/or analysed during the current study are available from the corresponding author on reasonable request.
